# Mycelia-Assisted Isolation of Non-Host Bacteria Able to Co-Transport Phages

**DOI:** 10.3390/v14020195

**Published:** 2022-01-20

**Authors:** Xin You, Niclas Klose, René Kallies, Hauke Harms, Antonis Chatzinotas, Lukas Y. Wick

**Affiliations:** 1 Helmholtz Centre for Environmental Research-UFZ, Department of Environmental Microbiology, Permoserstraße 15, 04318 Leipzig, Germany; xin.you@ufz.de (X.Y.); niclas.klose@ufz.de (N.K.); rene.kallies@ufz.de (R.K.); hauke.harms@ufz.de (H.H.); antonis.chatzinotas@ufz.de (A.C.); 2 German Centre for Integrative Biodiversity Research (iDiv) Halle-Jena-Leipzig, Puschstraße 4, 04103 Leipzig, Germany; 3 Institute of Biology, Leipzig University, Talstr.33, 04103 Leipzig, Germany

**Keywords:** viruses, co-transport, viral adsorption, motile bacteria, hitchhiking

## Abstract

Recent studies have demonstrated that phages can be co-transported with motile non-host bacteria, thereby enabling their invasion of biofilms and control of biofilm composition. Here, we developed a novel approach to isolate non-host bacteria able to co-transport phages from soil. It is based on the capability of phage-carrying non-host bacteria to move along mycelia out of soil and form colonies in plaques of their co-transported phages. The approach was tested using two model phages of differing surface hydrophobicity, i.e., hydrophobic *Escherichia* virus T4 (T4) and hydrophilic *Pseudoalteromonas* phage HS2 (HS2). The phages were mixed into soil and allowed to be transported by soil bacteria along the mycelia of *Pythium ultimum*. Five phage-carrying bacterial species were isolated (*Viridibacillus* sp., *Enterobacter* sp., *Serratia* sp., *Bacillus* sp., *Janthinobacterium* sp.). These bacteria exhibited phage adsorption efficiencies of ≈90–95% for hydrophobic T4 and 30–95% for hydrophilic HS2. The phage adsorption efficiency of *Viridibacillus* sp. was ≈95% for both phages and twofold higher than T4-or HS2-adsorption to their respective hosts, qualifying *Viridibacillus* sp. as a potential super carrier for phages. Our approach offers an effective and target-specific way to identify and isolate phage-carrying bacteria in natural and man-made environments.

## 1. Introduction

Bacteriophages (also termed phages) have received increasing interest as drivers of microbial ecology [1] and/or as agents to control bacterial biofilms relevant for human health [2,3], food hygiene [4,5], and environmental safety [6,7]. Phages are intrinsically non-motile anti-bacterial agents that typically disperse by diffusion (diffusion ≈ 4 × 10^−10^ m s^−1^ [8,9]) or advection to their hosts. Alternatively, phages may also adsorbed to (bio-) surfaces and disperse together with their vectors (e.g., non-host bacteria [10] or invertebrates [11]). At short distances, they may be ejected by explosive cell lysis [12], while phages immobilized e.g., on soil particles can act as set-and-wait predators for dispersing host bacteria in soil [13,14]. Hence, phage mobility is strongly influenced by interaction with their surrounding environments. In biofilms, phage mobility is the key determinant of its anti-bacterial efficiency [15] but is often hindered by adsorption to the biofilm extracellular matrix [16,17,18] (as reviewed by [19]). Using non-host motile bacteria as phage carriers, recent studies have demonstrated that lytic phages can be delivered to host biofilms by co-transport with motile bacteria [10] and exert anti-microbial effects on biofilm-dwelling bacteria [10,20,21]. Thus, co-transported phages may serve as “weapons” [22] and allow invasion of their carrier bacteria into host biofilms [10]. Phage co-transport with motile bacteria may also simulate other biological invasion processes (e.g., pests or pathogens being co-transported by other species [23,24,25]) and be useful in models challenging new hypotheses regarding biological invasion [10,26]. Although phage adsorption to environmental bacteria has been well described by multiple methods (e.g., metagenomic analysis [20], AdsorpSeq [27], fluorescent labeling [21], and cryo-electron microscopy [28]), few attempts have been made to isolate culturable phage-carrying bacteria as potential tools for challenging invasion hypothesis in microbial ecology or as phage-associated biofilm control agents in biotechnology.

Mycelia have been found as scaffolds for bacterial translocation (“fungal highway” [29]) in manifold microbial habitats including soil [29,30,31,32], plants [33,34], food [35,36], and human tissues [37]. Among such habitats, soil is a major reservoir for bacterial genetic diversity [38] and harbors abundant motile bacteria [30]. Motile soil bacteria are well-known vectors for inter-microbial co-transport (e.g., reviewed by [39]). However, much less is known about their ability to co-transport phages. Although often enabling bacterial dispersal through water-unsaturated zones, mycelia can retain the waterborne transport of phages [40,41]. Phage adsorption to (bio-)surfaces is mainly driven by the properties (e.g., hydrophobicity [40,42,43], surface charge [44]) of the phages and their interacting surfaces [20,40,45,46], phage morphology, and size [47] as well as environmental factors [48,49].

Here, we developed a novel laboratory approach to isolate non-host bacteria able to co-transport phages from soil. It is based on the observation that phages can interact with non-host bacteria and that phage-carrying bacteria can move along mycelia out of soil and develop separable colonies in plaques formed by their co-transported phages in host biofilms. We chose two model phages of differing surface hydrophobicity (i.e., hydrophobic *Escherichia* virus T4 (T4) and hydrophilic *Pseudoalteromonas* phage HS2 (HS2)), as hydrophobic phages are often found to be more adsorptive to (bio-)surfaces than hydrophilic phages [40,43]. To validate our system, we first mixed T4 or HS2 with gfp-labeled *Pseudomonas putida* KT2440 as inoculum and screened for plaques containing green fluorescent colonies that formed during the mycelia-assisted colonization of phage-carrying *P. putida* KT2440 in biofilms of the respective phages’ host strains. In a second step, we mixed model phages into fresh soil and let phage-carrying soil bacteria move out of the soil along mycelia and utilize phages as “weapons” to form colonies for direct isolation. We thereby isolated five bacterial strains that can co-transport phages (i.e., *Viridibacillus* sp., *Enterobacter* sp., *Serratia* sp., *Bacillus* sp. *Janthinobacterium* sp.) with the *Viridibacillus* sp. being a super carrier (≈95% adsorption efficiency for both hydrophobic T4 and hydrophilic HS2). Our approach offers an effective and target-specific way for the isolation of phage-carrying non-host bacteria. Such isolates do not only improve our knowledge on the transport and ecology of phages in natural (e.g., soil) ecosystem, but they may also be applied as biotechnological tools (together with phages) to control biofilms in natural and engineered ecosystems.

## 2. Materials and Methods

### 2.1. Strains, Growth Conditions, and Phage Enumeration

Two well-characterized lytic phages of distinct surface hydrophobicity were used as model phages (cf. Table S1), i.e., *Escherichia* virus T4 (NCBI:txid2681598) and *Pseudoalteromonas* phage HS2 [50] (NCBI:txid1348399). T4 has a hydrophobic surface with a water contact angle (*θ*_w_) of ≈95°, while HS2 is less hydrophobic (*θ*_w_ ≈ 40°) [47,51] (cf. Table S1). Both phages were propagated with their hosts using a liquid propagation method [52]. In short, infectious phages were added to host cultures (cf. Table S3) at an early exponential phase (OD_600_ ≈ 0.1) to have a multiplicity of infection of ≈0.1. The suspensions were further incubated at 22 °C for *Pseudoalteromonas* H13-15 and 30 °C for *E. coli* with 125 rpm until complete bacterial lysis was observed after 6–8 h of co-incubation. The lysed bacterial culture containing the propagated phages was further purified by centrifugation and filtration, as described earlier [47]. Freshly propagated phage solutions contained 10^11^–10^12^ plaque-forming units (PFUs) mL^−1^ and were stored at 4 °C as stock solutions. PFU enumeration was done using a whole-plate plaque assay as detailed earlier [53], allowing the double-layer counting plates to be incubated overnight at RT (30 °C for T4). The gfp-labeled *Pseudomonas putida* KT2440 (*P. putida* KT2440) was used as a model phage-carrying bacterium to validate our microcosm setup for phage-bacterial co-transport. The strain was kindly provided by Arnaud Dechesne (Technical University of Denmark). It was cultivated in LB medium with 150 rpm at 30 °C. The hyphae of the oomycete *Pythium ultimum* (*P. ultimum*) were used as model dispersal networks, as they are fast growing and allow for efficient bacterial dispersal [29]. *P. ultimum* was cultivated on potato dextrose agar (PDA) at room temperature (RT; approximately 22 °C) [54].

### 2.2. Description of the Microcosm Setup

**Figure 1:**
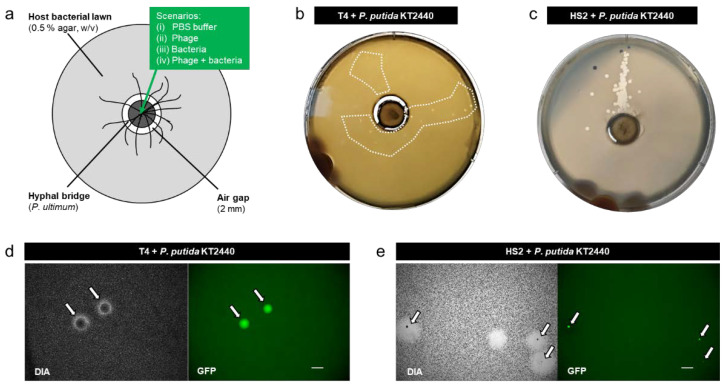
Schematic view of the microcosm used to isolate phage-carrying non-host bacteria (**a**), representative photographs of the microcosms after 1 d in the presence of T4 + *P. putida* KT2440 (**b**), and HS2 + *P. putida* KT2440; The dashed inserts in Figure 1b,c depict areas on plates containing turbid plaques.(**c**). Figure 1 (**d**). Representative micrograph of fluorescent colonies of co-transported *P. putida* KT2440 (indicated by white arrows and shown in green color under gfp channel) in the middle of turbid plaques of T4 (**d**) and HS2 (**e**) observed after 1 d on the respective host bacterial lawns. Scale bars reflect a length of 1 mm.

**Figure 2:**
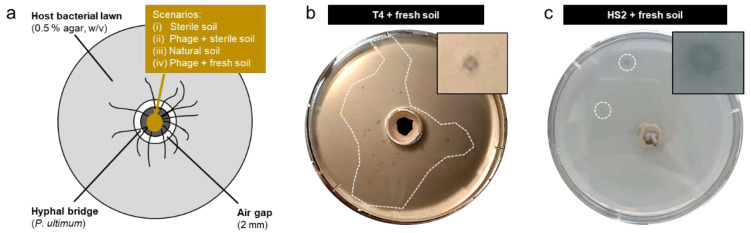
Schematic view of the microcosm used to isolate phage carrying non-host bacteria from fresh soil (**a**) and representative photographs of the microcosms after 1 d from “T4 in fresh soil” (**b**; cf. Figure S2 for control scenarios) and “HS2 in fresh soil” co-transport experiments (**c**). The dashed inserts in Figure 2b,c depict areas on plates containing turbid plaques.

To isolate phage-carrying bacteria, we developed a microbial model system on standard Petri dishes (diameter, ⌀ = 90 mm) allowing phage-carrying bacteria to disperse along mycelia and form colonies in plaques of their co-transported phages for direct isolation. The model system consisted of two agar patches: an inner circular agar patch (PDA, 1.5% agar (*w*/*v*), ⌀ = 12 mm) and an outer donut-shaped host agar ring (⌀ = 14–90 mm) that left a 2 mm airgap to the inner circle (Figure 1a and Figure 2a). The outer ring consisted of a double-layered agar: a top layer (0.5% agar (*w*/*v*), h = 1 mm) inoculated with the appropriate host bacteria (density: 5 ± 0.5 (SD) × 10^4^ CFU cm^−2^) and a bottom layer (1.5% agar (*w*/*v*), h = 0.5 cm) of the respective medium for phage host (cf. Table S3). The inner agar patch was pre-inoculated with *P. ultimum* 3–4 days prior to the final assembly of the microcosms, allowing >0.5 cm hyphal growth. The host-inoculated agar ring was freshly prepared using host bacterial culture (OD_600_ ≈ 0.4–0.6), as detailed by [55] and placed with around an inner agar patch with pre-grown *P. ultimum*, bridging the airgap on the day of the experiment. Assembled microcosms were covered with lids to minimize moisture loss before inoculation. For more details, cf. the extended protocol for microcosm assembly in the Supplementary Materials.

### 2.3. Validation of the Setup Using gfp-Labeled Model Bacteria

To test the suitability of the microcosm, we used flagellated and gfp-labeled *P. putida* KT2440 as carriers for the co-transport of T4 and HS2. This strain has been used as a model organism for bacterial dispersal along mycelia [56,57] and was shown to co-transport phages along mycelia into biofilms [10]. It can be recognized and visualized in mixed communities by its gfp label [58]. Four different inoculation scenarios were tested by adding 2.5 µL of phosphate-buffered saline (PBS, 100 mM, cf. Table S3) containing: (i) no bacteria and no phages (PBS), (ii) T4 or HS2 phages only, (iii) *P. putida* KT2440 only, or (iv) *P. putida* KT2440 in combination with T4 or HS2. The respective inocula (i.e., ≈10^8^ bacteria or ≈1.5 × 10^6^ phages) were placed in the center of the inner agar patch, and each scenario was performed in 4–8 replicates. To prepare the inoculum of scenario (iv), T4 or HS2 (≈6 × 10^9^ PFU mL^−1^) was co-incubated with *P. putida* KT2440 (≈8 × 10^9^ cells mL^−1^) in PBS at RT for 1 h in a 15 mL centrifuge tube at 100 rpm. The suspension was subsequently centrifuged (8000× *g* for 10 min at 4 °C) to discard free phages in the supernatant. The remaining pellet containing bacteria and adsorbed phages was resuspended in PBS by pipetting up and down and gently inverting the tube 4–6 times to obtain an OD_600_ ≈ 2. Then, the suspension was centrifuged (8000× *g* for 10 min at 4 °C), the supernatant was discarded, and the washed pellet was re-suspended in PBS to obtain a concentrated inoculum of calculated OD_600_ of ≈50. After inoculation, the Petri dishes were sealed with Parafilm (Bemis Company, Inc., Nina, WI, USA), placed in a plastic container, and incubated at 22 °C (or at 30 °C for T4) in the dark. After 1 day of incubation, the plates were screened for plaques and the formation of *P. putida* KT2440 colonies. Colonies of gfp-tagged *P. putida* KT2440 were confirmed with an epifluorescence microscope equipped with a black-and-white camera (AZ 100 Multizoom; Nikon, Amsterdam, The Netherlands) under the gfp channel using Nikon’s NIS Elements software. Plaques were also purified and tested against the respective hosts by performing plaque-forming assays (either *E. coli* or *Pseudoalteromonas* H13-15) to confirm their origin.

### 2.4. Isolation and Cultivation of Phage-Carrier Bacteria from Soil

To isolate phage-carrier bacteria from soil, phage-treated soil as inoculum was placed in the center of the inner agar patch. To do this, fresh topsoil (5–20 cm below the litter layer) was collected from a forest site (UTM: E 598830, N 5662818) in the Hainich Critical Zone Exploratory (Hainich CZE) in Thuringia, Germany and sieved through a 1 mm mesh. Then, 5 g of the sieved soil was placed into a 15 mL conical centrifuge tube together with 1 mL of a phage stock solution (≈10^10^ PFU mL^−1^) and 9 mL PBS to form a phage–soil suspension. The phage–soil suspension was further vortexed for 20 min at maximum speed and incubated for 3 h at 125 rpm (RT) to ensure the sufficient adsorption of phages to soil bacteria. After incubation, the phage–soil suspension was centrifuged (8000× *g* for 10 min at 4 °C), and free phages in the supernatant were discarded. A portion of the remaining pellet (⌀ < 8 mm) containing soil and soil bacteria with adsorbed phages was directly scooped out and placed on the round agar patch. Inocula with (i) (autoclaved) sterile soil, (ii) T4 or HS2 and sterile soil, and (iii) T4 or HS2 and fresh soil were tested. Each scenario was performed in 4–8 replicates. Sterile soil was obtained by twice autoclaving at 120 °C for 1 h, leaving about one day between the two sterilization steps. After inoculation, the Petri dishes were sealed with Parafilm, placed in a plastic container, and incubated at 22 °C (or at 30 °C for T4) in the dark. After 1 day, the plates were screened for plaques and colonies of soil bacteria (expected to appear in the plaques). Screened colonies (40 in total) in phage plaques were streaked by an inoculation loop and transferred on a new plate to form single colonies. Representative colonies of different morphologies were picked and purified by transferring to new plates for three times. They were further tested for phage susceptibility (spot test as described by [59]) to exclude potential contaminations from the phage host bacteria.

### 2.5. Identification and Characterization of Phage-Carrying Bacteria

The taxonomic affiliations of phage-insusceptible isolates (23 in total) were determined by PCR amplification of bacterial 16S rRNA gene fragments using the universal primer pairs 27F/1492R [60,61]. Paired-end sequencing of the obtained PCR product was done by GATC services (Eurofins, Ebersberg, Germany) using primers 27F/1492R (Biomers, Ulm, Germany). Isolates showing >99.9% identical sequences were classified as identical. Sequences of different bacteria were assigned to their closest reference relative using BLASTn [62] and cross-referenced with SILVA Incremental Aligner [63]. After taxonomic identification, the surface properties of all bacteria isolates were characterized by measuring *θ*_w_ and approximating their zeta potentials (*ζ*), as described previously [47,64]. Swimming and swarming motility assays were also performed as detailed by [56,65] to evaluate bacterial motility.

### 2.6. Phage Adsorption Assays

To determine the phage adsorption efficiencies of the soil isolates, phage adsorption assays were performed. All adsorption assays were performed at phage-to-bacteria ratios of 1 in triplicates, as described earlier [66]. In brief, suspensions of bacteria and phages (≈10^7^ CFU/PFU mL^−1^) were incubated in PBS at 22 °C for 1 h and centrifuged at 8000× *g* at 4 °C to pellet bacteria and adsorbed phages. For experiments comparing phage adsorption efficiencies to host and non-host bacteria (i.e., *Viridibacillus* sp.), suspensions of bacteria and phages were also incubated for 1 min, 5 min, and 15 min before centrifugation and evaluation. Amounts of adsorbed phages were estimated by the loss of free phages in the supernatant after centrifugation; i.e., phage adsorption (%) was calculated by the ratio of adsorbed phages to total phages prior to centrifugation. A phage-only control was included to determine adsorption efficiency. The colloidal stability of phage-only controls could be maintained to ≈2 h at RT and was not influenced by the centrifugation process (cf. Table S1).

## 3. Results

### 3.1. Microcosms for Isolation of Non-Host Bacteria Able to Co-Transport Phages

Microcosms were established, allowing the dispersal of phage-carrying bacteria along mycelia to a lawn of host bacteria that was spatially separated by a 2 mm air gap (Figure 1a). Consistent with our previous findings (e.g., [10,29,67]), the microcosm without fungal mycelia showed no transport of bacteria and/or phages over the airgap. We evaluated the transport of bacteria and/or phages along mycelia in four scenarios: (i) PBS, (ii) T4 or HS2, (iii) *P. putida* KT2440, and (iv) T4 + *P. putida* KT2440 or HS2 + *P. putida* KT2440. No plaques were observed in scenario (i) and scenario (ii) at any time (Figure S1), with the latter indicating no transport of phages along mycelia in the absence of bacteria. After 18 h and in accordance with our previous findings (e.g., [10,56]), we observed a dispersal of *P. putida* KT2440 along the hyphae (Figure S3a) in scenario (iii). However, no colonies of *P. putida* KT2440 were found in the host bacterial lawn on the outer agar ring, suggesting that *P. putida* KT 2440 was able to transport along mycelia but not able to establish in the pre-established lawn of host bacteria. When *P. putida* KT2440 had pre-adsorbed phages (scenario iv), we observed turbid plaques with distinct gfp-expressing colonies in the host bacterial lawns (Figure 1b,c), suggesting the successful co-transport and colonization of *P. putida* KT2440 in phage plaques (Figure 1d,e). Such turbid plaques were not observed at any time in our control scenarios (i), (ii), and (iii). Due to the small plaque size of T4, the turbid plaques of co-transported T4 and *P. putida* KT2440 were only visible on day 1. After that, *P. putida* KT2440 overgrew the area of the plaques as visualized by epifluorescence microscopy (Figure S3). Plaques formed by HS2 in *Pseudoalteromonas* bacterial lawns were bigger than T4 plaques (Figure 1b,c). Although this provided more space for *P. putida* KT2440, colonization with gfp-forming colonies was less explicit (Figure 1e and Figure S3), which was likely due to unfavorable growth conditions for strain KT2440 in the high-salinity *Pseudoalteromonas* medium (Table S3).

### 3.2. Isolation of Phage-Carrying Non-Host Bacteria from Soil

After validation of the system, we aimed to isolate phage-carrying bacteria from a pristine forest soil. To do so, we applied four different scenarios: (i) sterile soil, (ii) T4 or HS2 in sterile soil, (iii) fresh soil with no T4 or HS2, and (iv) T4 or HS2 in fresh soil (Figure 2a). No plaques or clearly distinguishable colonies were observed in scenarios (i), (ii), and (iii) at any time (Figure S2). However, when fresh soil had been augmented with phages (i.e., T4 or HS2), we observed turbid plaques with clearly separable colonies in lawns of host bacteria with high numbers of turbid plaques with T4 (Figure 2b) and only a few plaques with HS2 (Figure 2c). We conducted the “T4 in fresh soil” experiments using two more media suitable for *E. coli* (LB and R2A, cf. Table S3) to expand the range of soil bacteria able to establish in *E. coli*-cleared plaques. In total, we isolated 23 phage-insusceptible colonies that could be attributed to five distinct bacterial genera by 16S rRNA gene analysis: *Enterobacter* sp. and *Serratia* sp. from T4 plaques on DSM544; *Viridibacillus* sp. from T4 plaques on LB; *Janthinobacterium* sp. from T4 plaques on R2A; and *Bacillus* sp. from HS2 plaques on ZoBell (Table 1). We observed a clear influence of the bacterial growth medium on the isolates. For instance, the use of LB or R2A instead of DSM544 in T4 experiments resulted in the successful isolation of two additional phage-carrying bacteria (i.e., *Viridibacillus* sp. and *Janthinobacterium* sp., Table 1).

### 3.3. Characterization of Phage-Carrying Isolates

The five isolates were further tested for their swimming and swarming motility, their physicochemical surface properties (as drivers of the strength of colloidal interactions [47]), and their efficiency to adsorb hydrophobic T4 and less hydrophobic HS2, respectively.

**Figure 3:**
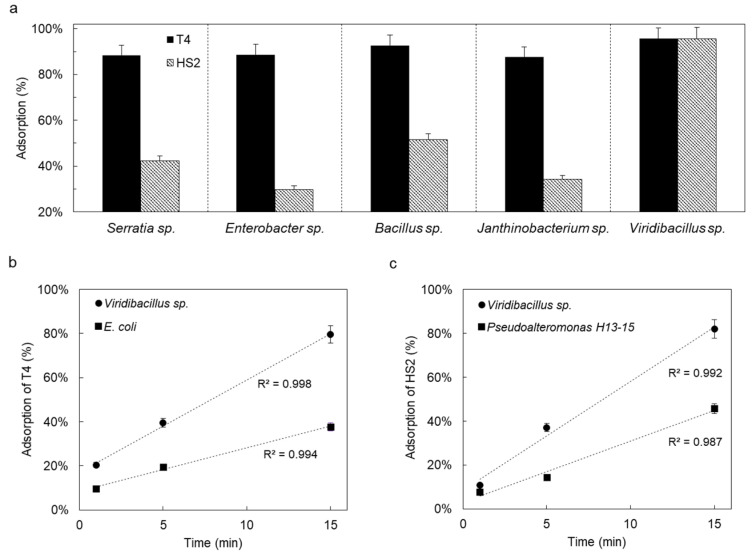
Phage adsorption efficiency (%) to isolated phage-carrying bacterial isolates (**a**) and comparison of time-dependent adsorption efficiencies of T4 (**b**) and HS2 (**c**) to *Viridibacillus* sp. and their respective hosts.

All bacteria were negatively charged with only slightly varying zeta potentials (−11 mV < *ζ* < −33 mV; Table 1). However, *Viridibacillus* sp. was significantly more hydrophobic (*θ*w ≈ 84°) than the other isolates (37° < *θ*_w_ < 62°, Table 1) and, hence, prone to strong interactions with other colloidal particles. *Viridibacillus* sp., *Enterobacter* sp., and *Serratia* sp. showed high swimming (⌀ > 77 mm d^−1^) and swarming (⌀ > 19 mm d^−1^) motility, while *Janthinobacterium* sp. and *Bacillus* sp. only exhibited weak (⌀ ~ 7 mm d^−1^) to intermediate (⌀ ~ 28 mm d^−1^) swimming motility. We observed high adsorption efficiencies (88–96%) of hydrophobic T4 to all five isolates (Figure 3a). Apart from *Viridibacillus* sp. (≈95%), the adsorption efficiencies of less hydrophobic HS2 were generally lower (30–52%, Figure 3) compared to the more hydrophobic T4. Given the efficient adsorption of T4 and HS2 to *Viridibacillus* sp., we further compared the adsorption kinetics of T4 and HS2 to their respective hosts with their adsorption to *Viridibacillus* sp. As shown in Figure 3b,c, adsorption by host bacteria and non-host *Viridibacillus* sp. both followed pseudo-first-order kinetics [68]. Phage adsorption to non-host *Viridibacillus* sp. thereby was approximately three times faster than to the respective phage hosts (c.f. Table S2), suggesting a surprisingly more efficient adsorption of T4 and HS2 to the non-host *Viridibacillus* strain.

## 4. Discussion

### 4.1. Mycelia-Assisted Isolation of Non-Host Bacteria Able to Co-Transport Phages

Although mycelia are well-described logistic networks for bacterial dispersal (“fungal highways” [29]), little is known about their effects on phage transport or phage-bacterial co-transport, even though such knowledge may help to resolve bacterial population dynamics in the hyphosphere [33,69] and put forward the hyphosphere as a model system to study biological invasions [10]. Here, we hypothesized that non-host bacteria can “pick up” phages and carry them along while dispersing along mycelia; this can be used for the targeted isolation of motile bacteria able to efficiently co-transport phages. Our hypothesis is based on previous knowledge on (i) the role of hyphae as conduits for bacterial dispersal (e.g., [29,31,35,67]), the observation that bacteria may serve as carriers of phages [20,70,71], and (iii) that co-transport of phages promotes the bacterial colonization of alien habitats (e.g., occupied by the phage’s hosts) [10,20,22,72]. In order to challenge our hypothesis, we prepared laboratory microcosms for selective mycelia-assisted isolation of phage-carrying bacteria and performed various control experiments. In the presence of mycelia yet in the absence of bacteria (scenario ii; cf. Results section), no plaques were observed in the spatially separated host bacterial lawn (Figure S1), suggesting an inefficient diffusion of phages along hyphae [9] or even hyphal phage retention [40,41]. Thus, mycelia played a decisive role for the bacterial co-transport of phages over airgaps. This is instrumental for the isolation of phage-carrying bacteria, as the sequential transport of (first) phage and (then) bacteria or co-infection [73,74] of non-interacting phages and bacteria would also lead to similar invasion effects into host biofilms. As biofilms typically are robust against colonization by competitors [10,20], we did not observe the colonization of transported *P. putida* KT2440 (validation experiment) or soil bacteria in the absence of adsorbed phages (Figures S1 and S2). In the presence of T4 or HS2 phages, both *P. putida* KT2440 and soil bacteria were able to colonize and grow in their respective host bacterial lawn (Figure 1b,c and Figure 2b,c) in competitor-free locations (i.e., plaques created by phages). Thus, mycelia-assisted transport allows isolating motile bacteria that can efficiently adsorb phages (Figure 3) and maintain their infectivity (Figure 1b,c and Figure 2b,c). As hyphae of *P. ultimum* unevenly bridged the air gap, we speculate that such irregular hyphal distribution may have influenced the transport of the bacterial shuttles and, hence, the observed distribution of plaques (e.g., Figure 1c) on the outer agar ring. Not all mycelial organisms may be used for mycelia-assisted isolation, as some fungi are known to antagonistically interact with bacteria [32]. We chose mycelia of *P. ultimum*, as they are hydrophilic [29] and known to be good dispersal networks for bacteria [10,29,75], exhibiting little negative interactions with bacteria. Mycelia that are known to be inhibited by phages, e.g., *Candida albicans* or *Aspergillus fumigatus* that exhibit inhibited growth in the presence of *Pseudomonas* phages [76,77] would likewise be not suitable for such isolation setups.

Higher adsorption of the more hydrophobic T4 than HS2 is in accordance with previous findings that hydrophobic phages (and other viruses) adsorb better to (bio-)surfaces than hydrophilic ones [40,42,43,47]. The hydrophobic surface of *Viridibacillus* sp. (composed of a unique S-layers with 40–60% hydrophobic amino acids [78]) exerted equally high attraction (≈95%) to both T4 and HS2. Overall, our data on phage adsorption emphasized the role of hydrophobic interactions for the interaction between phages and bacterial surfaces [79]. However, high adsorption efficiencies did not necessarily lead to efficient co-transport. For instance, *Viridibacillus* sp., although showing ≈95% adsorption efficiency of HS2, was not isolated as a HS2 carrier on ZoBell medium. We speculate that the high salinity of ZoBell medium selectively allowed (e.g., for *Bacillus* sp.) and hindered (e.g., for *Viridibacillus* sp.) the chemotaxis of HS2 co-transport bacteria. The faster adsorption of phages to *Viridibacillus* sp. than to their respective hosts might result from hydrophobic interaction [78] and/or the production of phage-adsorbing extracellular structures (e.g., [16,20]). As *Viridibacillus* sp. showed a strong adsorption of phages with differing surface hydrophobicity, future study of the cell surfaces of this strain may help to qualify it as a model bacteria for phage delivery in biotechnological applications.

### 4.2. Relevance for Phage Ecology and Biotechnology

Inter-microbial hitchhiking is a common strategy among soil microorganisms [39]. Although phage adsorption to environmental bacteria has been described by culture-independent techniques [20,21,27,28,80], few attempts have been made to isolate culturable bacteria able to co-transport phages. Using mycelia as pathways, we here isolated five typical soil bacteria [81,82] that can efficiently co-transport phages. While *Viridibacillus* and *Enterobacter* isolated from soil are frequently reported to be stress-tolerant and able to promote plant growth [83,84], their ability to co-transport phages may facilitate their invasion of and persistence in the rhizosphere. Dormant phages (i.e., prophages that are integrated into bacterial genomes) have been suggested to act as “bacterial weapons” (as reviewed by [22]). Although self-lysis to release phages can be a burden, prophage carrying bacteria are typically immune to their released phages and thus can trigger an epidemic among the susceptible competitors [82]. However, such competitors are normally restricted to strains of the same species [21]. Lytic phages interacting with non-host bacteria may further act as inter-species “weapons” for their bacterial carriers [10,20,21]. Hence, analyses on the ability of non-host bacteria to adsorb and transport phages may help to explain their environmental prevalence and become part of experimental phage ecology as e.g., recently proposed for testing new hypotheses of biological invasions [10]. Knowledge on phage-carrier bacteria will not only help to find novel non-host phage-carriers but also contribute to a better understanding of phage ecology and bacterial competition in natural and man-made environments. Non-host bacteria (i.e., *Bacillus cereus*) as phage carriers have been isolated and shown to facilitate phage invasion into biofilms and to mediate biofilm compositions in a (model) wastewater environment [20]. For water-unsaturated environments such as soil, phages usually get rapidly inactivated [85], which may hinder their anti-bacterial efficiency against unwanted biofilms (e.g., of plant pathogens). As phage adsorption to (bio-) surfaces [49,86] (e.g., with non-host bacteria [10,21]) is known to protect phages from inactivation, phage-carrying bacteria may also be used both to prolong phage infectivity and targeted phage delivery to biofilms in natural and man-made environments. For instance, *Viridibacillus* and *Enterobacter* sp. have been described as potential plant-growth-promoting bacteria [83,84]. Being also identified as good phage carriers in this study, they may also be considered as candidates for phage delivery in soil to combat unwanted biofilms of plant and root pathogens. However, further refinement of the method may be needed, as any cultivation-based selection and isolation of bacteria will depend on the nutrient media used in the host bacteria lawn. As hyphal-riding bacteria are similarly found in other environments than soil, future work may allow isolating new phage carriers of high relevance in phage ecology and biotechnology from other natural and man-made environments. In addition, our easy and inexpensive isolation approach may also become helpful in the education field, representing a very good task for laboratory practical courses in viral ecology.

## Figures and Tables

**Table 1:** Origin and characterization of phage-carrying bacteria isolated from forest soil.

Bacterial Isolates		Phys.-Chem. Surface Properties		Motility ^f^	
Co-transported phage	Next related sequence in NCBI	Zeta potential	Contact angle	Swimming	Swarming
(isolation medium)	(% similarity; Gram +/−)	(*ζ*, mV)	(*θ_w_*, degree)	(⌀, mm d^−1^)	(⌀, mm d^−1^)
T4 (DSM544)	*Serratia* sp. ^a^ (99.80%; Gram −)	−33 ± 1	59 ± 3	77 ± 5	23 ± 1
T4 (DSM544)	*Enterobacter* sp. ^b^ (99.86%; Gram −)	−13 ± 0	37 ± 2	86 ± 2	35 ± 2
T4 (LB)	*Viridibacillus* sp. ^c^ (99.93%; Gram +)	−23 ± 2	84 ± 2	85 ± 1	28 ± 3
T4 (R2A)	*Janthinobacterium* sp. ^d^ (99.93%; Gram −)	−14 ± 1	62 ± 6	28 ± 1	-
HS2 (ZoBell)	*Bacillus* sp. ^e^ (98.90%; Gram +)	−11 ± 1	42 ± 7	7 ± 1	-

^a–e^ NCBI accession numbers: ^a^ MT631995, ^b^ CP011591, ^c^ MH669125, ^d^ MF774161, ^e^ MW181146; ^f^ Motility was estimated by the average bacterial displacement in diameter (mm) per day (d^−1^) on swimming/swarming plates after incubation for 48 h at RT. For fast swimming isolates, only displacement within 24 h was used for estimation.

## Data Availability

Not applicable.

## Supplementary Materials

The following supporting information can be downloaded at https://www.mdpi.com/article/10.3390/v14020195/s1. Extended protocol for microcosm assembly. Figure S1: Representative images of microcosms of control scenarios (i.e., PBS, T4/HS2, and *P. putida* KT2440) in experiments to validate the setups using gfp-labeled *P. putida* KT2440 as phage carrier. Figure S2. Representative images of microcosms of control scenarios (i.e., “sterile soil”, “sterile soil + T4/HS2”, and “fresh soil”) in experiments to isolate soil bacteria able to co-transport phages. Figure S3. Representative fluorescent micrographs of dispersion of gfp-labeled *P. putida* KT2440 (i.e., without phages) along the hyphae after 18 h and colonization of gfp-labeled phage-carrying *P. putida* KT2440 on the outer host agar ring after 2 days. Table S1. Characteristics and colloidal stability of used model phages. Table S2. Time-dependent phage adsorption rate constant (*k*) in experiments comparing phage adsorption efficiencies to host and to non-host *Viridibacillus* sp. Table S3. Overview of buffers and media used in this study.
