# Supplementary material for: Mycelia-Assisted Isolation of Non-Host Bacteria Able to Co-Transport Phages
Source: Viruses. 2022 Jan 20;14(2):195. doi: 10.3390/v14020195 (PMC8877629; doi:10.3390/v14020195)
Supplement: Supplementary file 1 [file viruses-14-00195-s001.zip › viruses-1523599-supplementary.pdf]

Supporting information:

Mycelia-assisted isolation of non-host bacteria able to co-transport phages

Xin You[1], *Niclas Klose*[1], *René Kallies*[1], *Hauke Harms*[1,2], *Antonis Chatzinotas*[1,2,3], *Lukas Y. Wick*[1*]

[1] Helmholtz Centre for Environmental Research - UFZ, Department of Environmental Microbiology, Permoserstraße 15, 04318 Leipzig, Germany
[2] German Centre for Integrative Biodiversity Research (iDiv) Halle-Jena-Leipzig, Puschstraße 4, 04103 Leipzig, Germany
[3] Leipzig University, Institute of Biology, Talstr.33, Leipzig 04103, Germany

Keywords: viruses, co-transport, viral adsorption, motile bacteria, hitchhiking

Number of pages: 10
Number of figures: 3
Number of tables: 3

[*] Corresponding author. Phone: +49 341 235 1316, fax: +49 341 235 45 1316, e-mail: lukas.wick@ufz.de.

Extended protocol for microcosm assembly

Materials:

- Cork borer of nested size 9 and 11
- Tweezers (preferably type 7a with bent tips)
- Spatula with rectangular and flat ends (bent to ~60° at one end)
- Inoculation needles
- Squared paper
- Petri dishes (⌀ = 90 mm)
- PDA plate (1.5% w/v agar), DSM544 plate (1.5% w/v agar, c.f. Table S3), DSM544 soft agar tubes (5ml, 0.5% w/v agar)
- Freshly revived *P. ultimum* plates (PDA)
- Freshly prepared *E. coli* pre-culture in liquid DSM544 medium

Note: All reusable non-sterile tools needs to be exposed under UV for 30 min before each experiment and flamed shortly before use. Experiment needs to be conducted in sterile **laminar flow hood**.

1. Preparation of the inner fungal agar patch with *P. ultimum*

Note: This step should be conducted 3-4 days before the microcosm mounting to allow the mycelia to grow to > 5 cm off of the agar edge (slow-growing fungi might need longer time).

1.1 Use size 9 cork borer to cut agar patch from PDA plates and transfer the cut agar patch to empty Petri dishes by spatula. Each Petri dish should not host more than 5 agar pieces.

1.2 Arrange the agar patches in Petri dishes to have enough distance (>1 cm) between each other.

1.3 Use inoculum needles to scratch visible amount of mycelia from the *P. ultimum* plates and transfer them on the prepared agar patches as inoculum.

1.4 Seal the Petri dish with Parafilm (optional: place them in a humidified plastic container) and incubate at 25°C in the dark.

<u>Practical information</u>: The amount of the agar patches prepared should excess 1.5 to 2 times the number of patches needed. This is to ensure spare patch material as fungi sometimes fail to grow on some agar patches.

2. Preparation of the outer double-layer agar ring

Note: This step is to be conducted on the day of the experiment.

2.1 Transfer 10 µL *E. coli* pre-culture in 20 mL DSM544 liquid medium. Incubate at 37°C with 125 rpm for ~4 h until $OD_{600} \approx 0.4$.

2.2 Melt DSM544 soft agar by microwaving and incubate them at 55°C in water bath.

2.3 Allow the molten agar to cool (it should feel warm but not hot to touch by the back of your hand).

2.4 Transfer 100 µL *E. coli* culture ($OD_{600} \approx 0.4$) into the molten agar and vortex to mix.

2.5 Pour the culture onto a bottom agar (DSM544). Tilt the plate in all directions to ensure the soft agar evenly covers the bottom agar.

2.6 Cover the plate and allow it to solidify 10-20 min.

<u>Practical information</u>: If the soft agar tubes are not freshly prepared, add 0.5 mL liquid medium in each tube directly after melting and vortex to mix. This is to prevent excessive loss of moisture.

3. Microcosm mounting

Note: Check mycelial growth from 1.4 and select well-grown agar patches for mounting.

3.1 Use squared paper to prepare a mould for the microcosm as shown in Fig 1a.

3.2 Place the paper mould under the solidified *E. coli* double-layered agar plate.

3.3 Use size 11 cork borer to dig a hole in the middle of the *E. coli* plate.

3.4 Use a tweezer (preferably type 7a) to transfer the prepared *P. ultimum* agar patch into the center of the hole. Use the tweezer tips to grab from the bottom of the agar patch and avoid as much as possible to touch extended mycelia in the air.

<u>Practical information</u>: Transfer *P. ultimum* agar patch promptly, as mycelia of *P. ultimum* tend to shrink within a few seconds once picked up by tweezers. While placing the agar patch into the hole, try to tilt slightly the agar patch in the direction with highest volume of mycelia and let the mycelia touch the outer agar ring. This will make sure that mycelia bridge at least one direction between the inner agar patch and the outer agar ring.

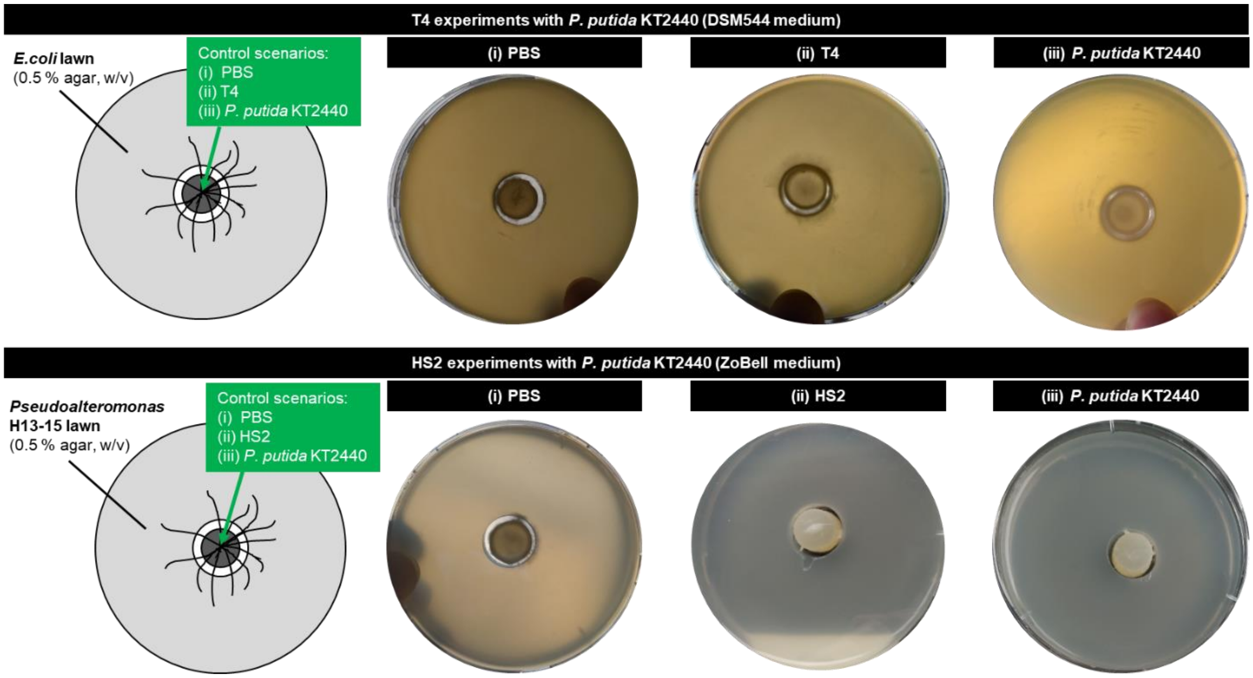

Figure S1. Representative images of microcosms of control scenarios (i.e. PBS, T4/HS2 and *P. putida* KT2440) in experiments to validate the setups using gfp-labelled *P. putida* KT2440 as phage carrier. Upper panel: control scenarios in T4 experiments with *P. putida* KT2440 on DSM544 medium after 1 day; Lower panel: control scenarios in HS2 experiments with *P. putida* KT2440 on ZoBell medium after 1 day.

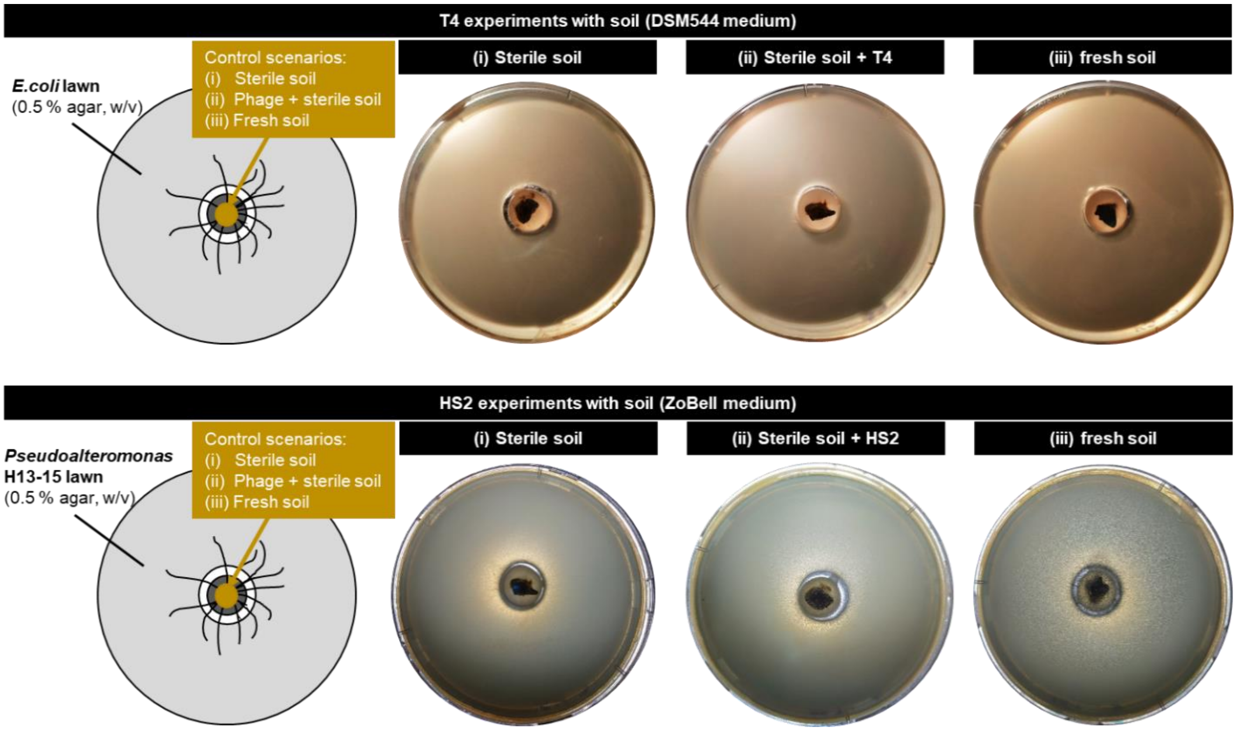

Figure S2. Representative images of microcosms of control scenarios (i.e. "sterile soil", "sterile soil + T4/HS2" and "fresh soil") in experiments to isolate soil bacteria able to co-transport phages. Upper panel: control scenarios in T4 experiments with soil on DSM544 medium after 1 day; Lower panel: control scenarios in HS2 experiments with soil on ZoBell medium after 1 day.

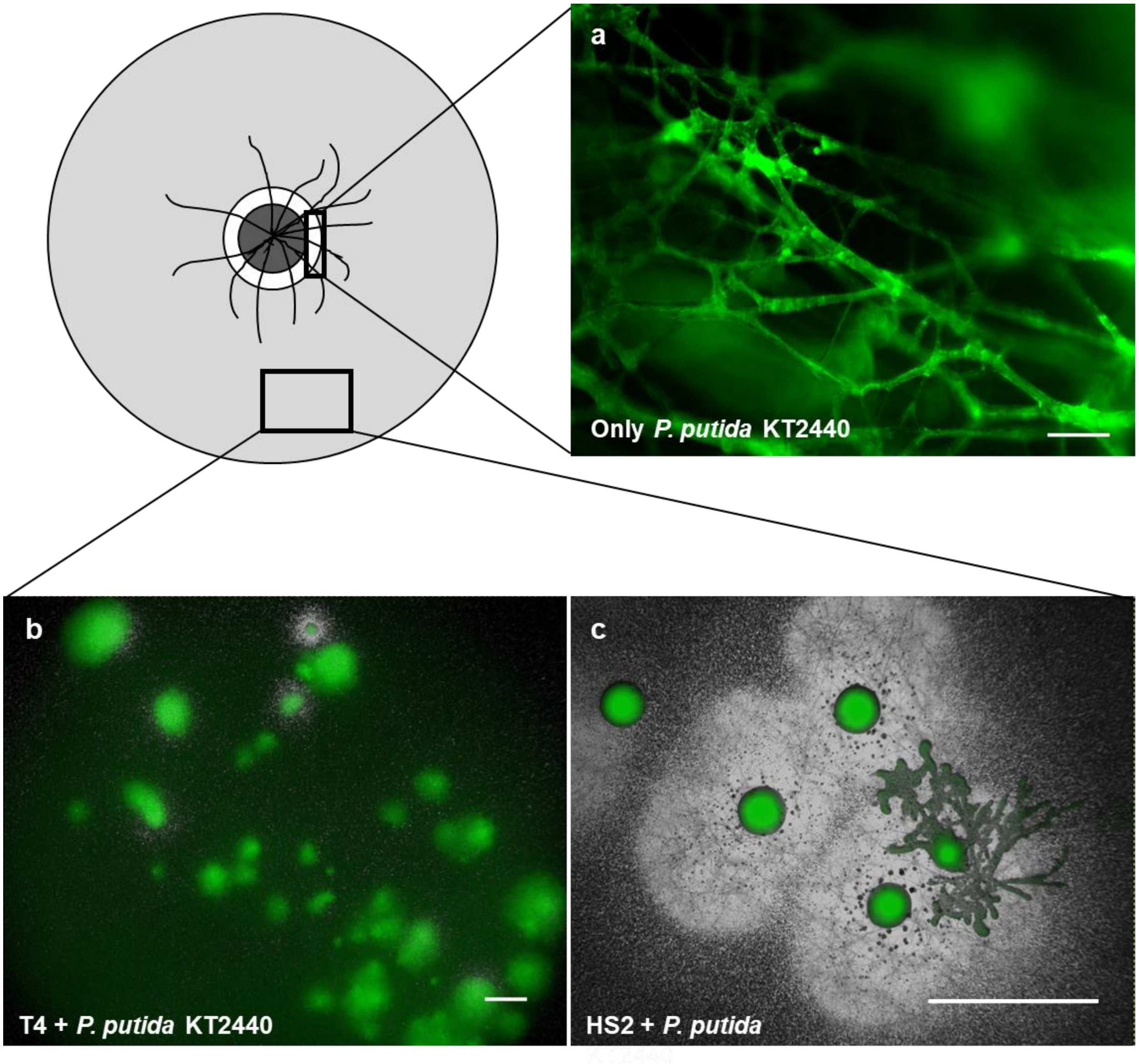

Figure S3. Representative fluorescent micrographs of dispersion of gfp-labelled *P. putida* KT2440 (i.e. without phages) along the hyphae after 18h and colonization of gfp-labelled phage-carrying *P. putida* KT2440 on the outer host agar ring after 2 days. **Fig S3a**. Representative fluorescent micrographs of dispersion of gfp-labelled *P. putida* KT2440 (i.e. without phages) along the hyphae over the air gap after 18h; **Fig S3b.** Colonization and over-growth of T4-carrying *P. putida* KT2440 in T4 plaques on *E.coli* biofilms after 2 days; **Fig S3c.** Colonization and growth of HS2-carrying *P. putida* KT2440 in HS2 plaques on *Pseudoalteromonas* H13-15 biofilms after 2 days. Scale bar represents a distance of 1 mm.

Table S1. Characteristics and colloidal stability of used model phages

Phage name & family	Phage host	Zeta potential	Water contact angle	Size (head/tail)	Colloidal stability [a]	
		ζ (mV)	Θ_w (degree)	(nm)	In buffer [b]	By centrifugation [c]
HS2 [1,2] *Siphoviridae*	*E. coli* (Migula 1895)	- 3 ± 1	40 ± 5 [3]	210 (60/150)	105 ± 9	104 ± 6
T4 *Myoviridae*	*Pseudoalteromonas H13-15*	-13 ± 1	95 ± 5 [3]	203 (90/113)	107 ± 4	96 ± 8

[a] Colloidal stability was estimated by the quotient of phage concentrations (PFU mL^{-1}) at the beginning to the end of a treatment; [b] Colloidal stability estimated by static incubation of phages in PBS for 2 h; [c] Colloidal stability estimated by centrifugation of phages suspensions at 8,000 x g at 4°C for 10 min.

Table S2. Time-dependent phage adsorption rate constant (k)[4] in experiments comparing phage adsorption efficiencies to host and to non-host *Viridibacillus* sp.

Bacteria	Phage	Time-dependent phage adsorption rate constant [a] (k, × 10^{-9} min^{-1})		
		1min	5min	15min
Viridibacillus sp.	T4	9.4 ± 3.5	4.0 ± 0.9	4.0 ± 0.5
	HS2	4.7 ± 2.2	3.8 ± 0.5	4.8 ± 0.9
E. coli	T4	4.2 ± 2.8	1.8 ± 0.1	1.3 ± 0.3
Pseudoalteromonas H13-15	HS2	3.4 ± 2.2	1.3 ± 0.5	1.7 ± 0.3

[a] Time-dependent phage adsorption rate constant (k) was determined based on Krueger (1931)[4]: $k = \ln(P_0/P_t)/(t \times B)$, where t is the incubation time (min), B the bacteria number (CFU), and P_0 and P_t PFUs of free phages at time 0 and t, respectively. Data were presented as average ± standard deviation (n=3).

Table S3. Overview of buffers and media used in this study

Buffer	Recipe (amounts added per 1 L of buffer)
PBS (100 mM)	0.87 g K_2HPO_4 L^{-1}, 0.68 g KH_2PO_4 L^{-1}
KNO_3 (10 mM)	1.01 g KNO_3 L^{-1}

Medium	Bacterial strain applied	Recipe (amounts added per 1 L of medium)
DSM 544	*E. coli* (Migula 1895)<br>*Enterobacter* sp.<br>*Serratia* sp.	10 g casein hydrolysate L^{-1}, 5 g NaCl L^{-1}, 1 g casamino acid L^{-1}, 5 g yeast extract L^{-1}, 2 g $MgSO_4 \times 7H_2O$, 2 g maltose L^{-1}
LB	*E. coli* (Migula 1895)<br>*Viridibacillus* sp.	20 g LB-Medium (Lennox) L^{-1}, 10 g tryptone L^{-1}, 5 g yeast extract L^{-1}, 5 g NaCl L^{-1}
R2A	*E. coli* (Migula 1895)<br>*Janthinobacterium* sp.	1 g caseine L^{-1}, 0.5 g yeast extract L^{-1}, 0,5 g glucose L^{-1}, 0.5 g starch L^{-1}, 0.3 g K_2HPO_4 L^{-1}, 0.024 g $MgSO_4$ L^{-1}, 0.3 g $C_3H_3NaO_3$ L^{-1}
ZoBell (i.e. 50% ZoBell 2216E)	*Pseudoalteromonas* H13-15<br>*Bacillus* sp.	2.5 g peptone L^{-1}, 0.5 g yeast extract L^{-1}, 26 g sea salts L^{-1}

References

1. Duhaime, M.B.; Solonenko, N.; Roux, S.; Verberkmoes, N.C.; Wichels, A.; Sullivan, M.B. Comparative Omics and Trait Analyses of Marine Pseudoalteromonas Phages Advance the Phage OTU Concept. *Front. Microbiol.* **2017**, *8*, 1241, doi:10.3389/fmicb.2017.01241.

2. Duhaime, M.B.; Wichels, A.; Sullivan, M.B. Six *Pseudoalteromonas* Strains Isolated from Surface Waters of Kabeltonne, Offshore Helgoland, North Sea. *Genome Announc.* **2016**, *4*, e01697-15, doi:10.1128/genomeA.01697-15.

3. Ghanem, N.; Kiesel, B.; Kallies, R.; Harms, H.; Chatzinotas, A.; Wick, L.Y. Marine Phages As Tracers: Effects of Size, Morphology, and Physico–Chemical Surface Properties on Transport in a Porous Medium. *Environ. Sci. Technol.* **2016**, *50*, 12816–12824, doi:10.1021/acs.est.6b04236.

4. Krueger, A.P. The Sorption of bacteriophage by living and dead susceptible bacteria : I. equilibirum conditions. *J. Gen. Physiol.* **1931**, *14*, 493–516, doi:10.1085/jgp.14.4.493.
